# Supplementary material for: An Efficient Continuous Flow Synthesis for the Preparation of N-Arylhydroxylamines: Via a DMAP-Mediated Hydrogenation Process
Source: Molecules. 2023 Mar 27;28(7):2968. doi: 10.3390/molecules28072968 (PMC10096002; doi:10.3390/molecules28072968)
Supplement: Supplementary file 1 [file molecules-28-02968-s001.zip › molecules-2305754-SI.pdf]

SUPPORTING INFORMATION

An Efficient Continuous Flow Synthesis for the Preparation of *N*-Arylhydroxylamines: Via a DMAP-Mediated Hydrogenation Process

Jianli Chen [1,2,*], Xinyu Lin [2], Feng Xu [3], Kejie Chai [2], Minna Ren [2], Zhiqun Yu [2], Weike Su [2] and Fengfan Liu [2,*]

[1] *College of New Materials Engineering, Jiaxing Nanhu University, Jiaxing 314000, China*

[2] *National Engineering Research Center for Process Development of Active Pharmaceutical Ingredients, Collaborative Innovation Center of Yangtze River Delta Region Green Pharmaceuticals, Zhejiang University of Technology, Hangzhou 310014, China*

[3] *Raybow (Hangzhou) Pharmaceutical Co., Ltd., Hangzhou 310014, China*

** Correspondence: 220163@jxnhu.edu.cn (J.C.); liufengfan@zjut.edu.cn (F.L.)*

Table of Content

Supplementary data for experiments······3
Data of products······6
Compounds spectra······8
References······21

Supplementary data for experiments

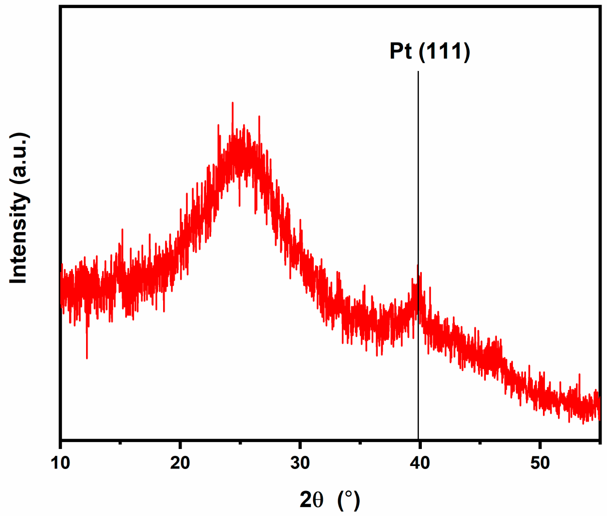

Figure S1. XRD diffractogram of 5 wt.% Pt/C

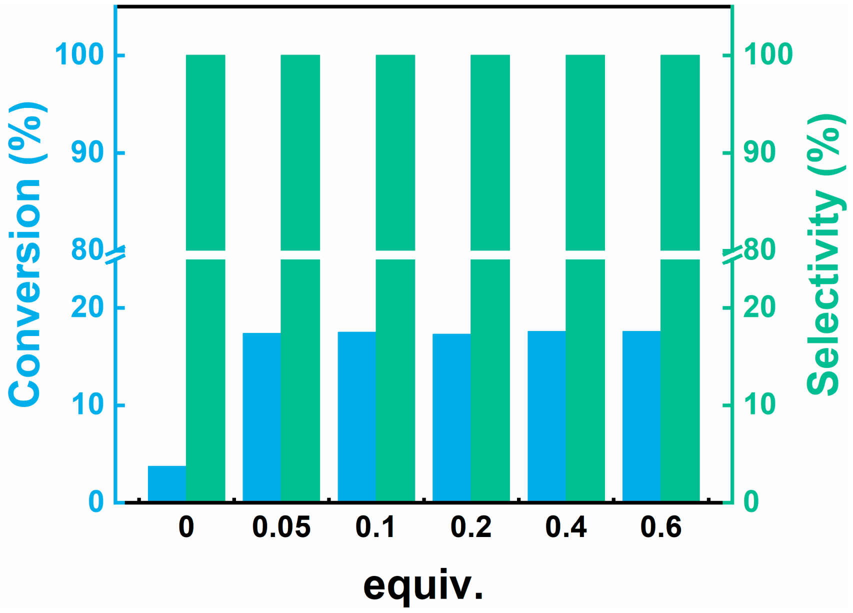

Figure S2. Inspection of DMAP equivalent in batch. Conditions: **1a** (0.78g), 5 wt. % Pt/C (5 mg), DMAP in THF (20mL), 303K, 600 rpm, 1.0 MPa H_2, 10 min reaction time.

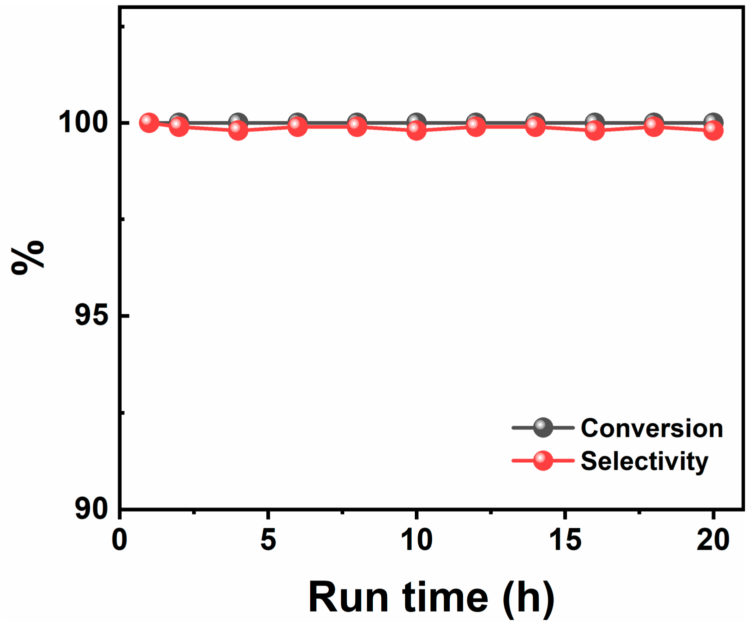

Figure S3. Run time of hydrogenation in flow. Conditions: 0.1 M solution of **1a** in THF, 5 wt.% Pt/C (1 cartridge; Ø3.0×50 mm; 0.1 g), H_2 pressure (6 bar), back pressure (4 bar), liquid flowrate (0.5 mL·min^{-1}), temperature (25 °C).

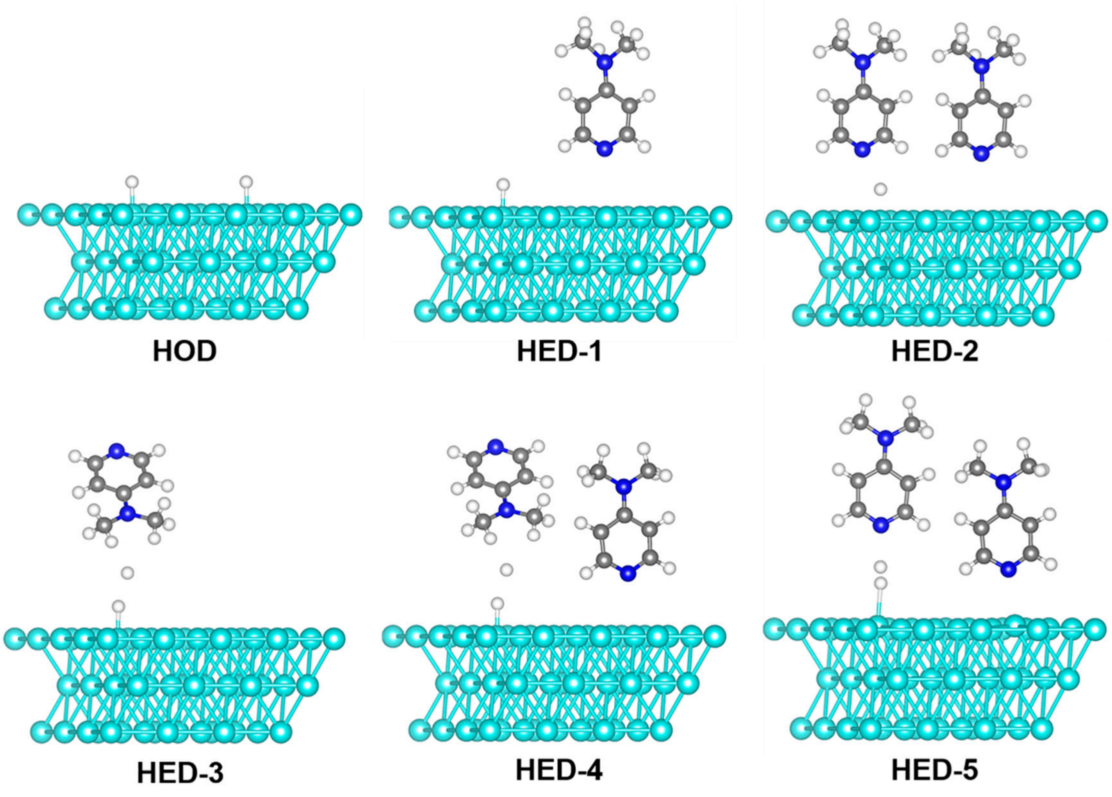

Figure S4. H_2 adsorption on the Pt surface. White, blue, and grey spheres depict H, N, and C atoms, respectively.

Table S1. Control experiments

Entry	Condition	Conv. (%)
1	No 5 wt% Pt/C	0
2	No H_2	0

Conditions: **1a** (0.78g), 5 wt. % Pt/C (5 mg), DMAP (0.078g) in THF (20mL), 303K, 600 rpm, 1.0 MPa H_2, 1 h reaction time.

Table S2. Bader charge analysis

	**Charge distribution (Q_{H1}, Q_{H2})**	
HOD	-0.13	-0.13
HED-1	-0.11	0.37
HED-2	-0.12	0.39
HED-3	-0.19	0.43
HED-4	/	/
HED-5	-0.09	0.33

Table S3. Investigation of TEA equivalent in batch

Entry	Equivalent of TEA	Conv. (%)
1	None	4
2	0.05 eq. TEA	15
3	0.1 eq. TEA	24

Conditions: **1a** (0.78g), 5 wt. % Pt/C (5 mg), DMAP (0.078g) in THF (20mL), 303K, 600 rpm, 1.0 MPa H_2, 10 min reaction time.

Data of products

***N*-(2-chlorophenyl)hydroxylamine (1b)**[1]

^{1}H NMR (600 MHz, DMSO-*d6*): 8.55 (s, 1H), 8.18 (s, 1H), 7.25-7.17 (m, 3H), 6.79-6.76 (td, *J* = 7.8, 1.8 Hz, 1H).
^{13}C NMR (150 MHz, DMSO-*d6*): 147.4, 128.4, 127.4, 119.7, 116.8, 114.0.

***N*-(4-chlorophenyl)hydroxylamine (2b)**[1]

^{1}H NMR (600 MHz, DMSO-*d6*): 8.43 (s, 1H), 8.39 (s, 1H), 7.20-7.18 (d, *J* = 8.4 Hz, 2H), 6.85-6.84 (d, *J* = 8.4 Hz, 2H).
^{13}C NMR (150 MHz, DMSO-*d6*): 151.0, 128.3, 122.5, 114.4.

***N*-(3-chlorophenyl)hydroxylamine (3b)**[2]

^{1}H NMR (600 MHz, DMSO-*d6*): 8.51 (s, 1H), 8.49 (s, 1H), 7.17-7.14 (t, J = 7.8 Hz, 1H), 6.85 (s, 1H), 6.76-6.74 (dd, *J* = 7.8, 1.8 Hz, 2H).
^{13}C NMR (150 MHz, DMSO-*d6*): 153.7, 133.3, 130.0, 118.6, 112.1, 111.4.

***N*-(2-bromophenyl)hydroxylamine (4b)**[2]

^{1}H NMR (600 MHz, DMSO-*d6*): 8.58 (s, 1H), 7.97 (s, 1H), 7.41-7.40 (dd, *J* = 7.8, 1.2 Hz, 1H), 7.28-7.25 (m, 1H), 7.17-7.15 (dd, *J* = 8.4, 1.8 Hz, 1H), 6.74-6.71 (td, *J* = 7.2, 1.2 Hz, 1H).
^{13}C NMR (150 MHz, DMSO-*d6*): 148.6, 131.8, 128.2, 120.7, 114.6, 106.7.

***N*-(2-iodophenyl)hydroxylamine (5b)**[3]

^{1}H NMR (600 MHz, DMSO-*d6*): 8.59 (s, 1H), 7.64-7.62 (m, 2H), 7.30-7.27 (m, 1H), 7.11-7.09 (dd, *J* = 8.4, 1.8 Hz, 1H), 6.61-6.58 (td, *J* = 7.2, 1.8 Hz, 1H).
^{13}C NMR (150 MHz, DMSO-*d6*): 150.9, 138.2, 128.8, 121.6, 114.2, 81.6.

ethyl 2-(hydroxyamino)benzoate (6b)[2]

^{1}H NMR (600 MHz, DMSO-*d6*): 8.95 (s, 1H), 8.66(s, 1H), 7.79-7.77 (d, *J* = 9.0 Hz, 2H), 6.85-6.84 (d, *J* = 9.0 Hz, 2H), 3.76 (s, 3H).
^{13}C NMR (150 MHz, DMSO-*d6*): 166.3, 156.0, 130.5, 119.2, 111.3, 51.5.

1-(4-(hydroxyamino)phenyl)ethanone (7b)[4]

^{1}H NMR (600 MHz, DMSO-*d6*): 8.99 (s, 1H), 8.69 (s, 1H), 7.80-7.79 (d, *J* = 9.0 Hz, 2H), 6.84-6.83 (d, *J* = 9.0 Hz, 2H), 2.44 (s, 3H).
^{13}C NMR (150 MHz, DMSO-*d6*): 195.8, 156.0, 129.9, 127.8, 111.0, 26.1.

N-phenylhydroxylamine (8b)[1]

^{1}H NMR (600 MHz, DMSO-d_6): 8.28 (s, 1H), 8.23 (s, 1H), 7.18-7.15 (t, *J* = 7.8 Hz, 2H), 6.86-6.84 (d, *J* = 8.4 Hz, 2H), 6.76-6.73 (t, *J* = 7.8 Hz, 1H).
^{13}C NMR (150 MHz, DMSO-d_6): 152.1, 128.4, 119.2, 113.0.

N-(2-tolyl)hydroxylamine (9b)[1]

^{1}H NMR (600 MHz, DMSO-d_6): 8.22 (s, 1H), 7.90 (s, 1H), 7.11-7.09 (m, 2H), 6.98-6.97 (d, *J* = 7.2 Hz, 1H), 6.72-6.69 (td, *J* = 6.6, 2.4 Hz, 1H), 2.08 (s, 3H).
^{13}C NMR (150 MHz, DMSO-d_6): 149.7, 129.4, 126.3, 121.9, 119.1, 112.1, 16.9.

N-(2-(((1-(4-chlorophenyl)-1H-pyrazol-3-yl)oxy)methyl)phenyl)hydroxylamine (10b)[5]

^{1}H NMR (600 MHz, DMSO-d_6): 8.40 (s, 1H), 8.37 (d, *J* = 3.0 Hz, 1H), 8.15 (s, 1H), 7.78-7.77 (d, *J* = 9.0 Hz, 2H), 7.52-7.50 (d, *J* = 9.0 Hz, 2H), 7.32-7.31(d, *J* = 6.0 Hz, 1H), 7.26-7.23 (t, *J* = 7.8 Hz, 1H), 7.20-7.19 (d, *J* = 7.8 Hz, 1H), 6.83-6.80 (t, *J* = 7.8 Hz, 1H), 6.09 (d, *J* = 3.0 Hz, 1H), 5.21 (s, 2H).
^{13}C NMR (150 MHz, DMSO-d_6): 163.9, 149.5, 138.5, 129.5, 129.3, 129.1, 128.8, 128.6, 121.0, 119.2, 118.7, 112.9, 94.7, 67.0.

o-Chloroaniline (1c)[1]

^{1}H NMR (600 MHz, DMSO-d_6): 7.17-7.16 (dd, *J* = 7.8, 1.2 Hz, 1H), 7.02-6.99 (td, *J* = 6.0, 1.8 Hz, 1H), 6.80-6.78 (dd, *J* = 8.4, 1.8 Hz, 1H), 6.54-6.51 (td, *J* = 7.2, 1.2 Hz, 1H), 5.29 (s, 2H).
^{13}C NMR (150 MHz, DMSO-d_6): 144.6, 129.0, 127.6, 117.1, 116.8, 115.4.

Aniline (1d)[1]

^{1}H NMR (600 MHz, DMSO-d_6): 7.02-7.00 (t, *J* = 15.6 Hz, 2H), 6.8-6.56 (dd, *J* = 8.4, 1.2 Hz, 2H), 6.51-6.48 (t, *J* =14.4 Hz, 1H), 4.98 (s, 2H).
^{13}C NMR (150 MHz, DMSO-d_6): 148.6, 128.8, 115.7, 113.9.

Compounds spectra

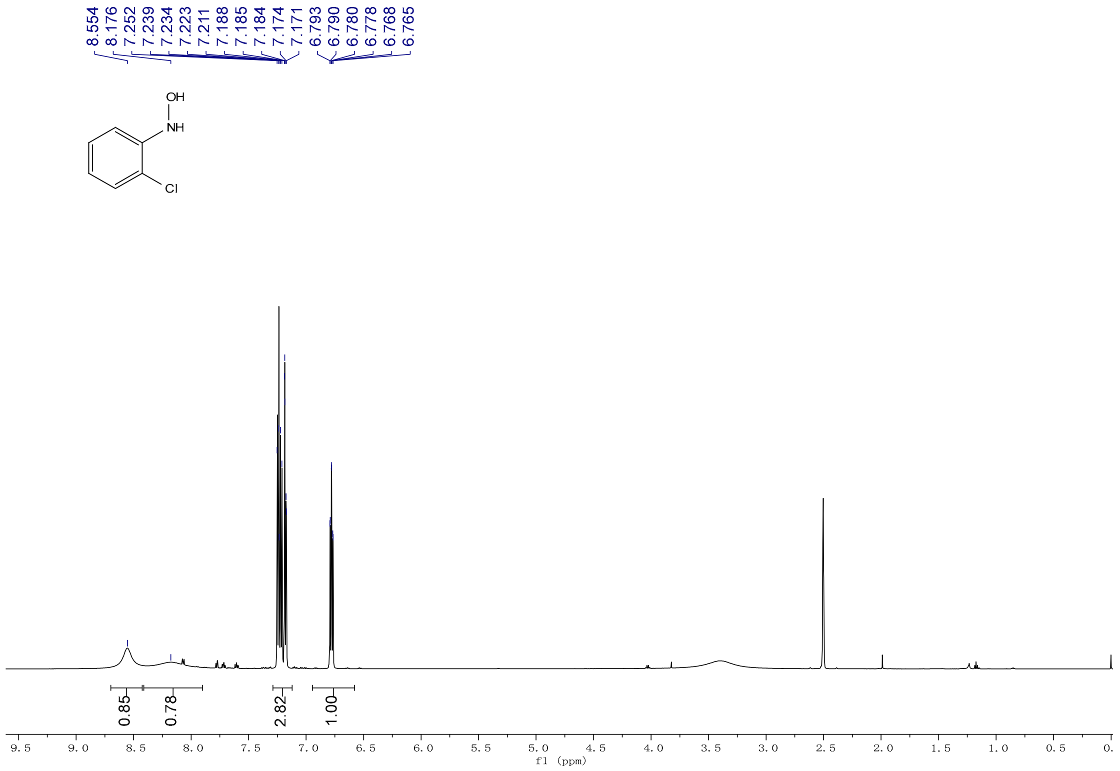

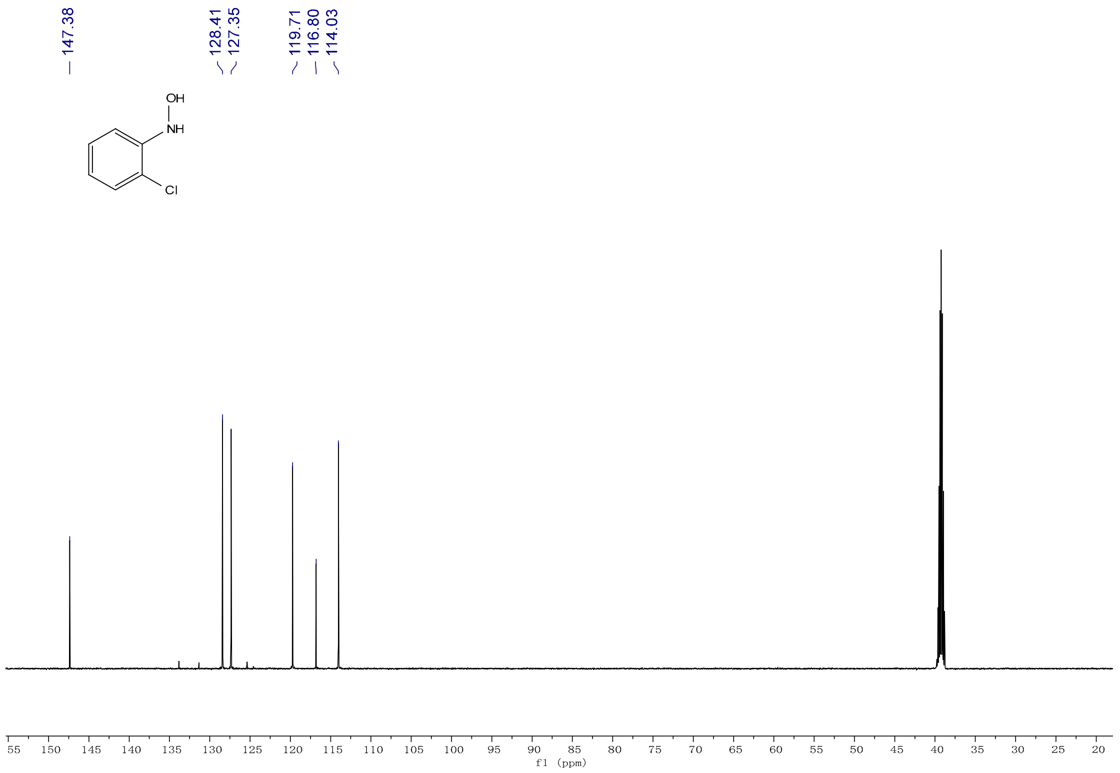

Figure S5. ^{1}H and ^{13}C NMR spectra of *N*-(2-chlorophenyl)hydroxylamine

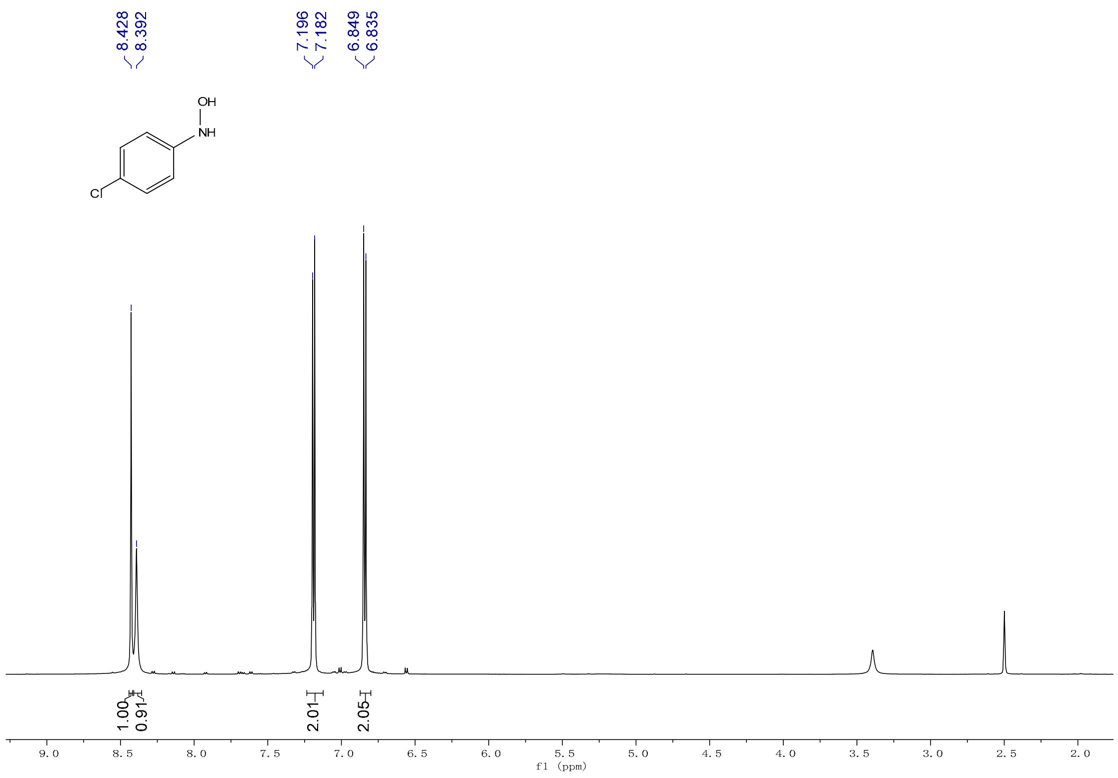

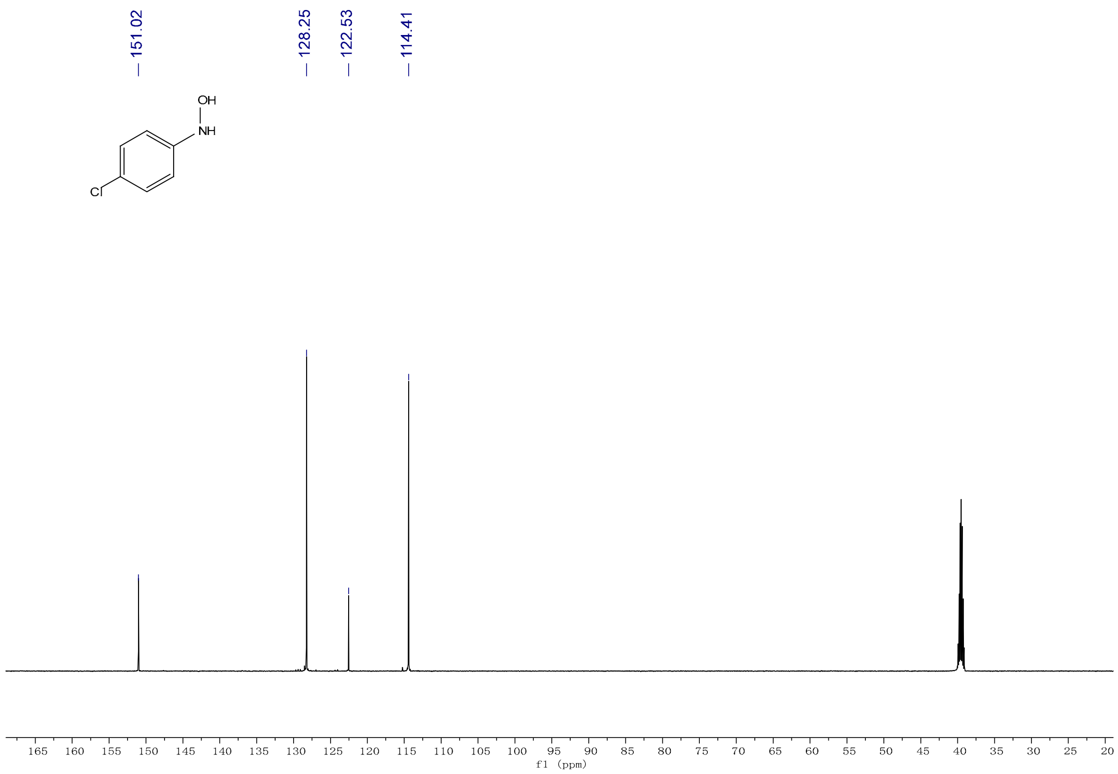

Figure S6. ^{1}H and ^{13}C NMR spectra of ***N*-(4-chlorophenyl)hydroxylamine**

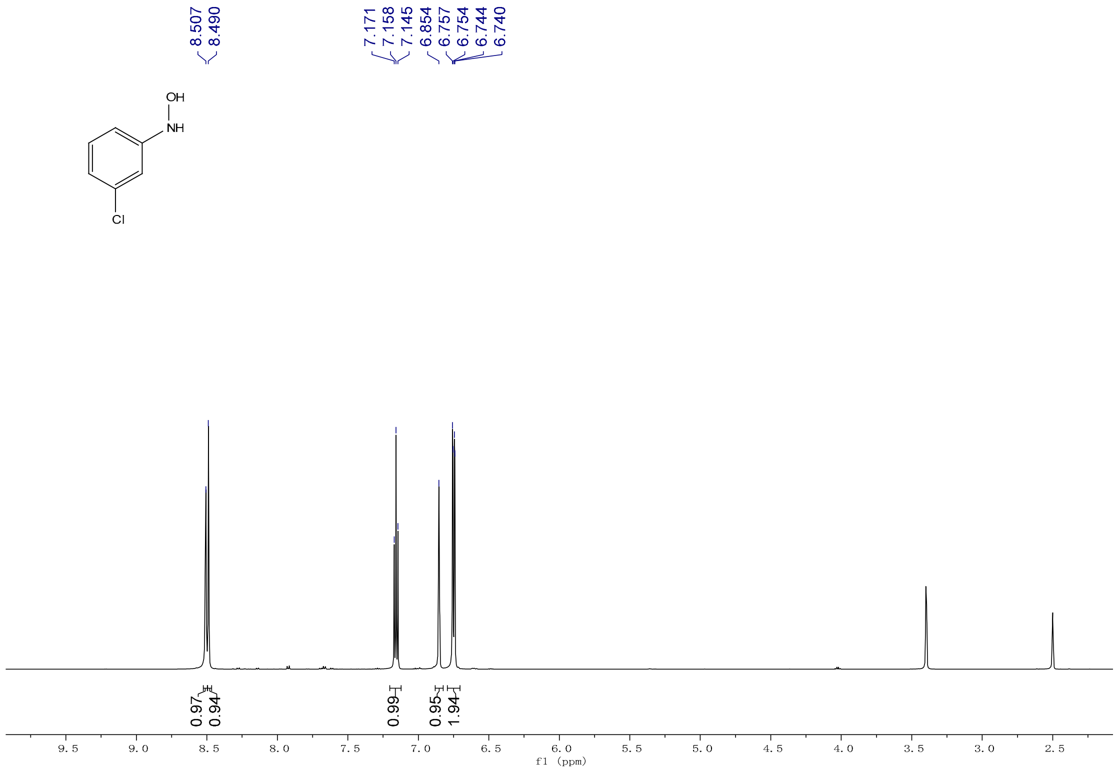

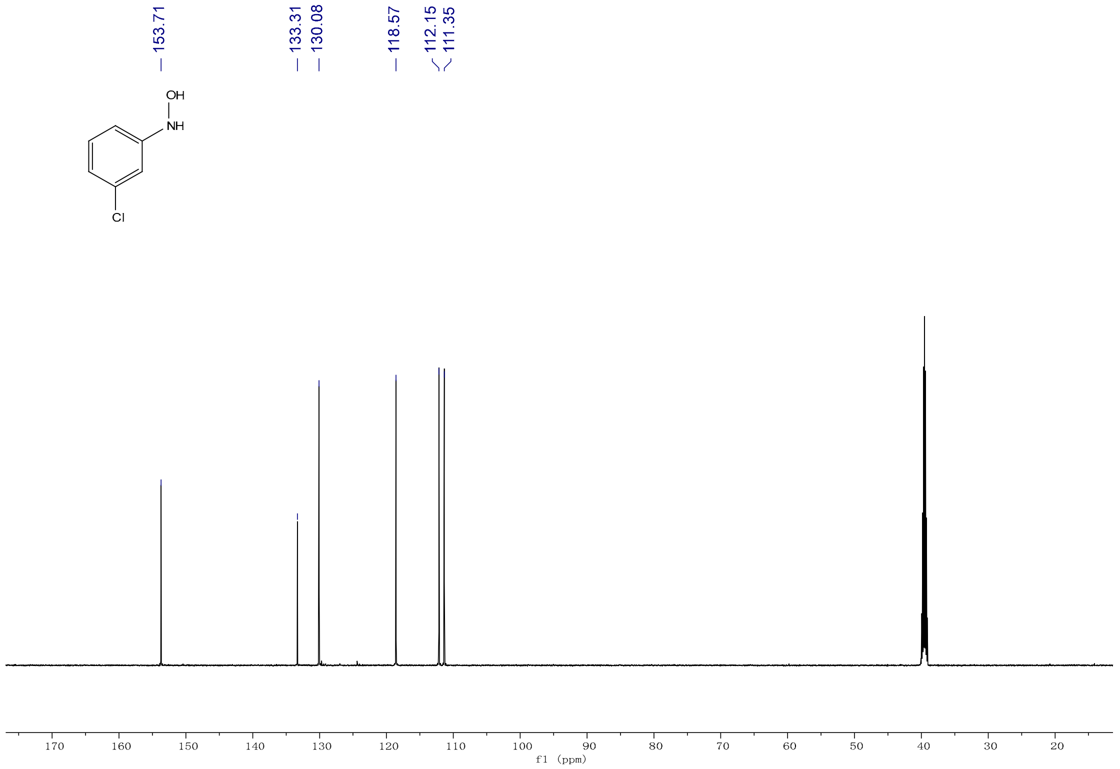

Figure S7. ^{1}H and ^{13}C NMR spectra of *N*-(3-chlorophenyl)hydroxylamine

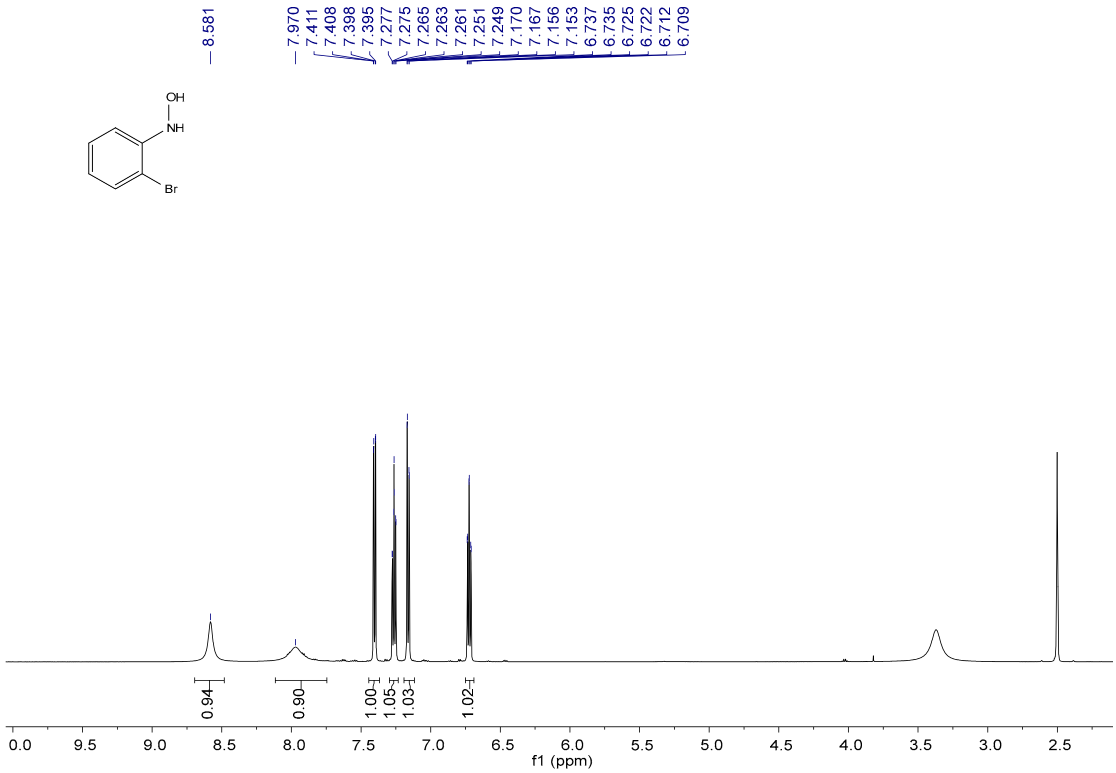

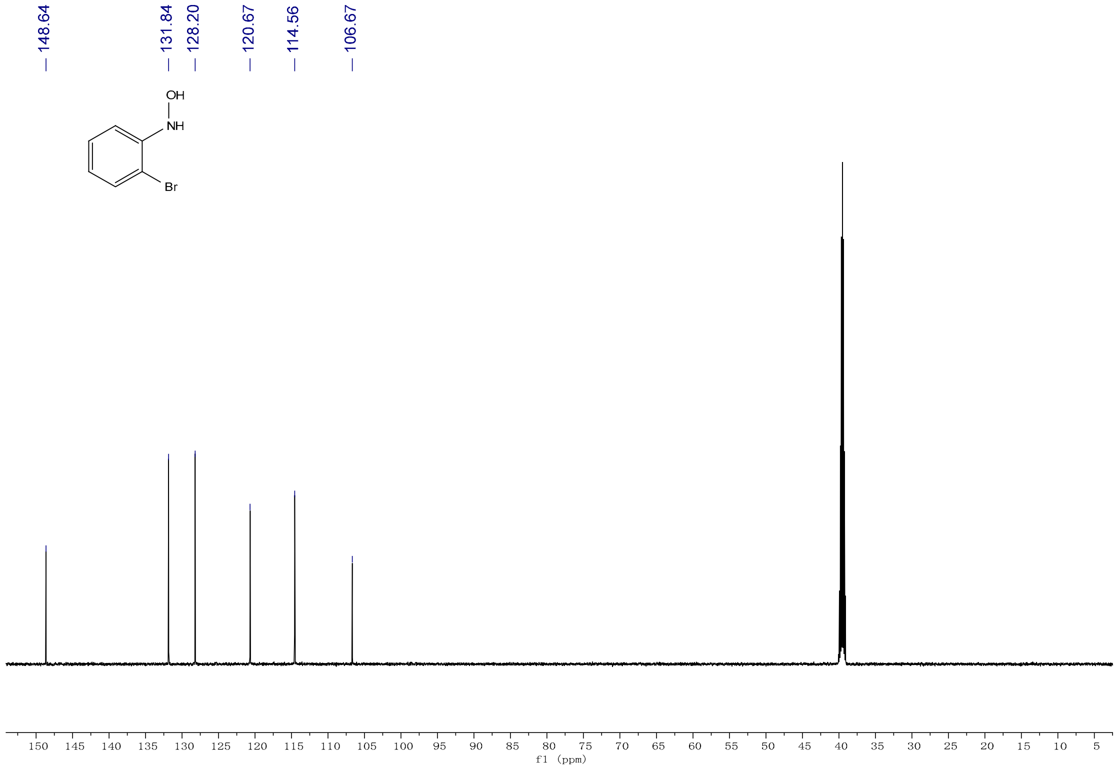

Figure S8. ^{1}H and ^{13}C **NMR spectra of *N*-(2-bromophenyl)hydroxylamine**

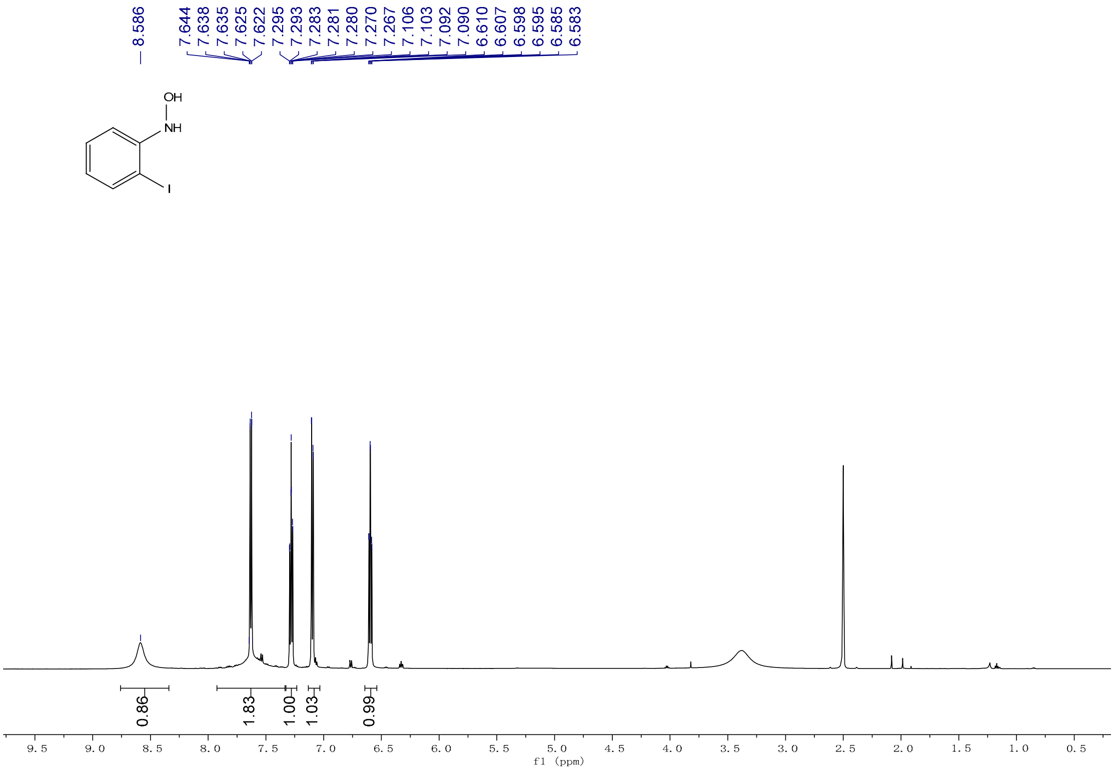

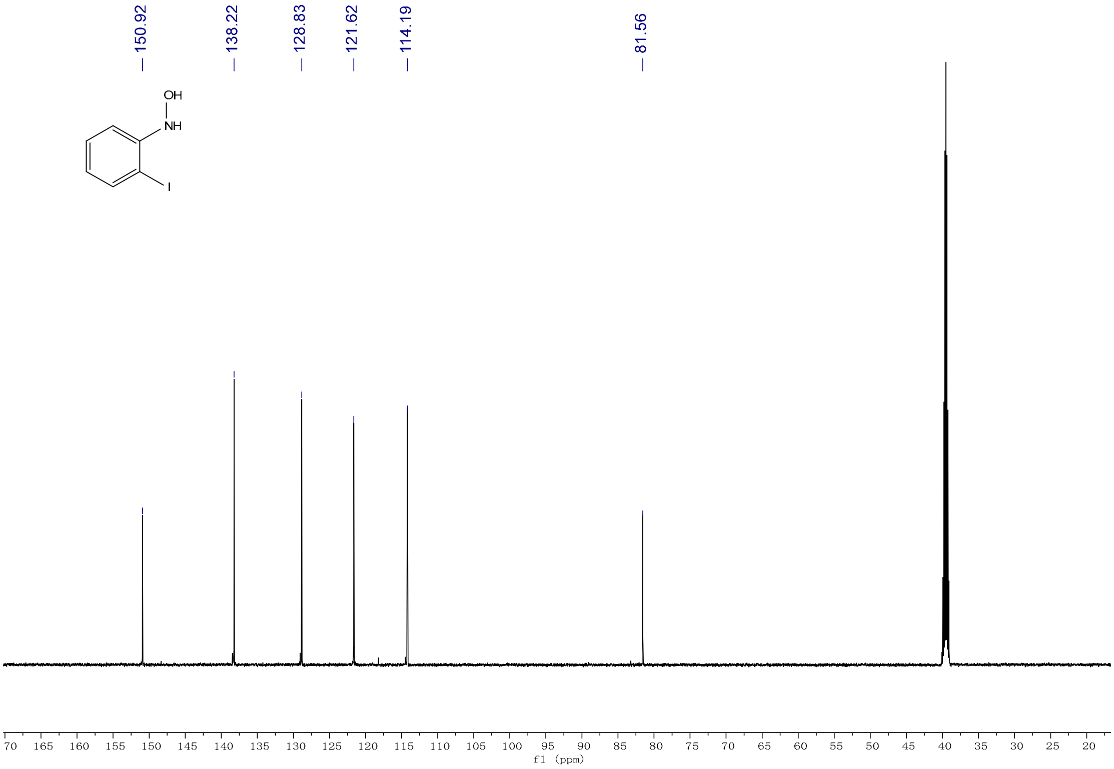

Figure S9. ^{1}H **and** ^{13}C **NMR spectra of *N*-(2-iodophenyl)hydroxylamine**

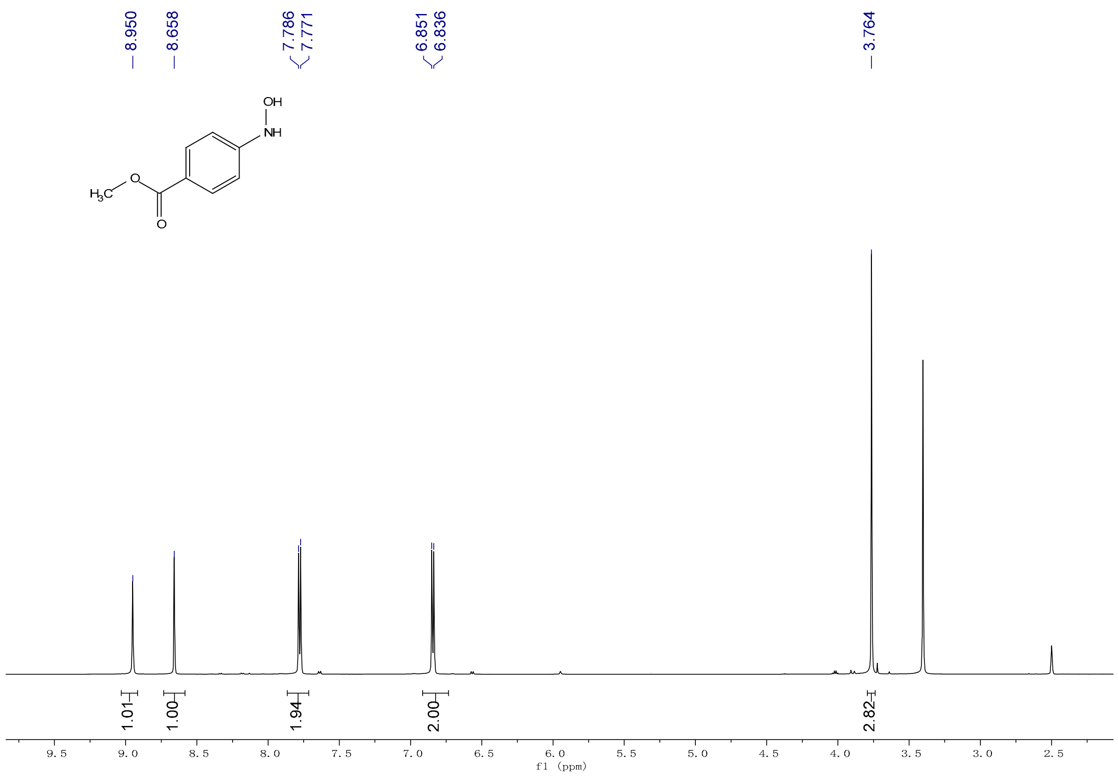

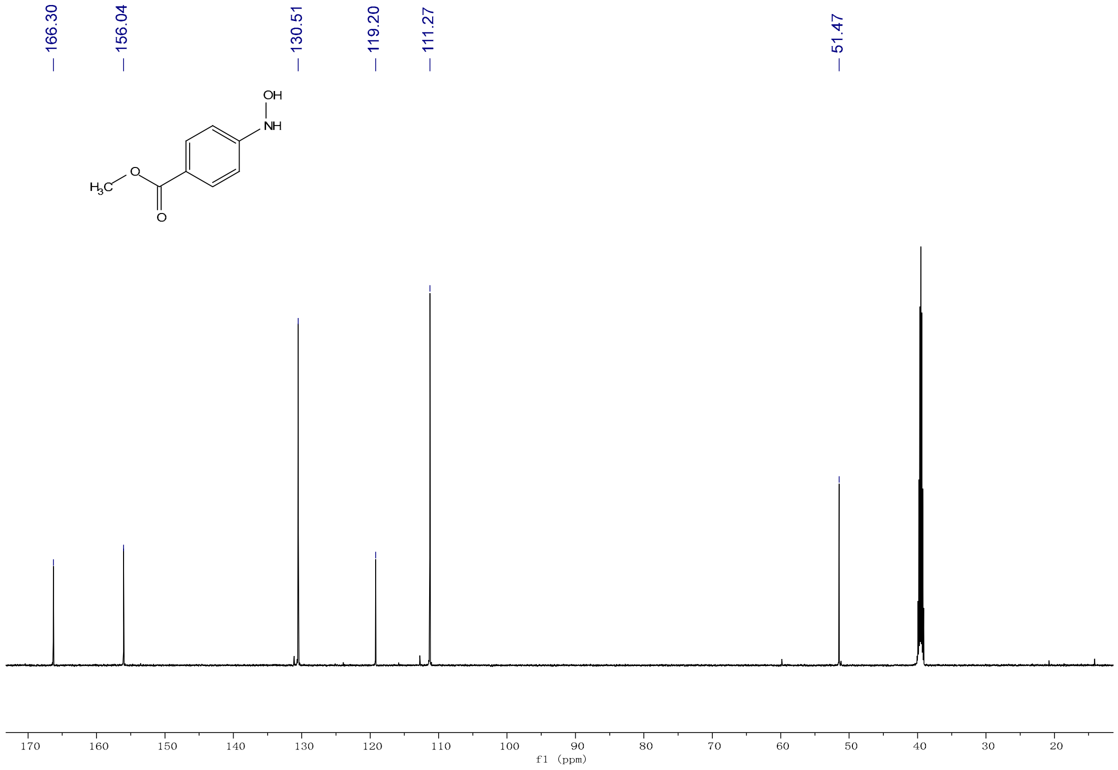

Figure S10. ^{1}H and ^{13}C NMR spectra of ethyl 2-(hydroxyamino)benzoate

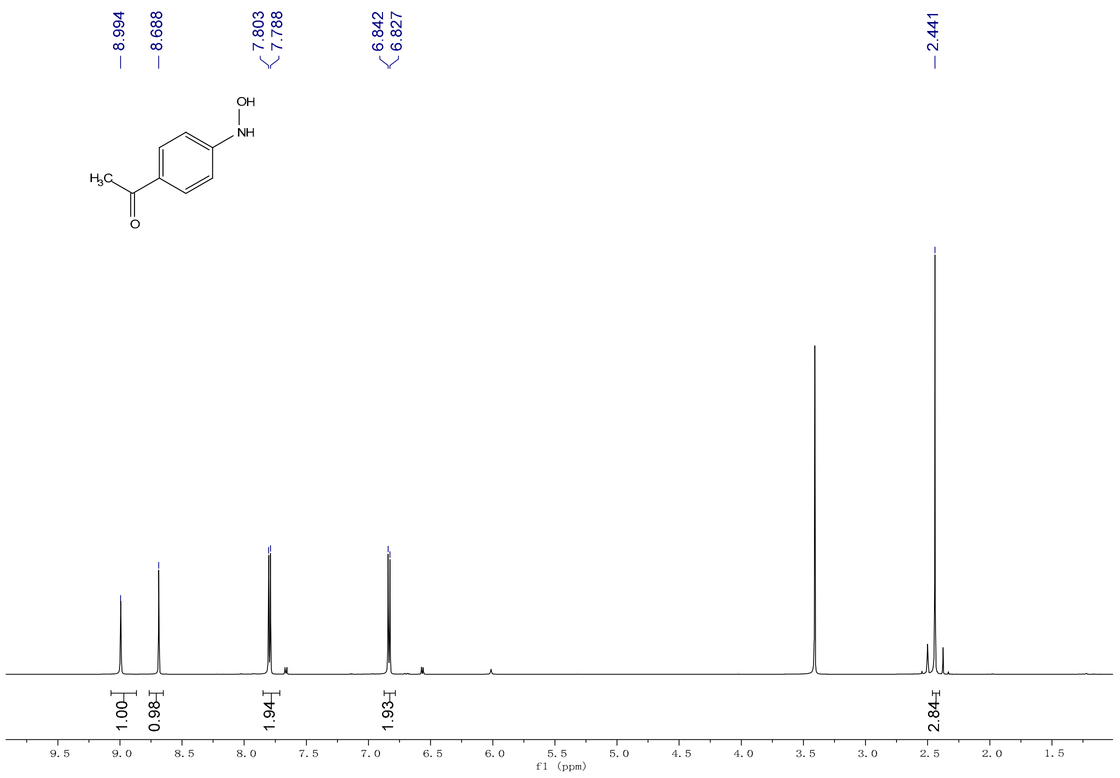

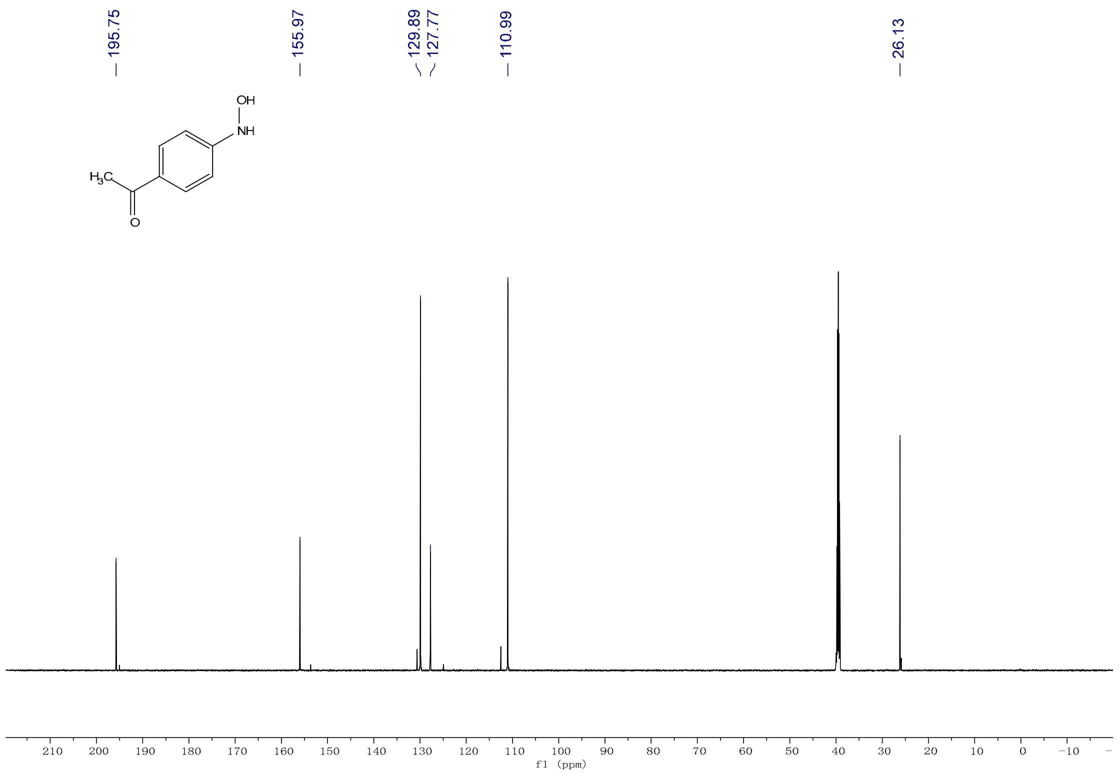

Figure S11. ^{1}H and ^{13}C NMR spectra of **1-(4-(hydroxyamino)phenyl)ethanone**

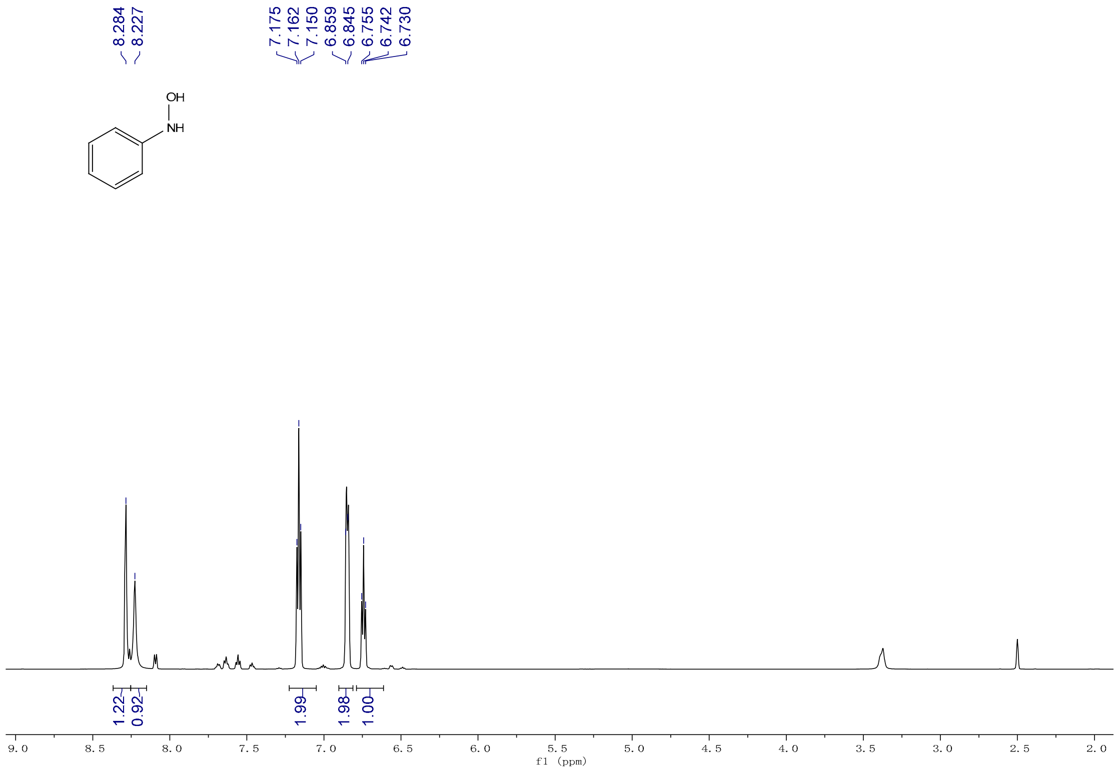

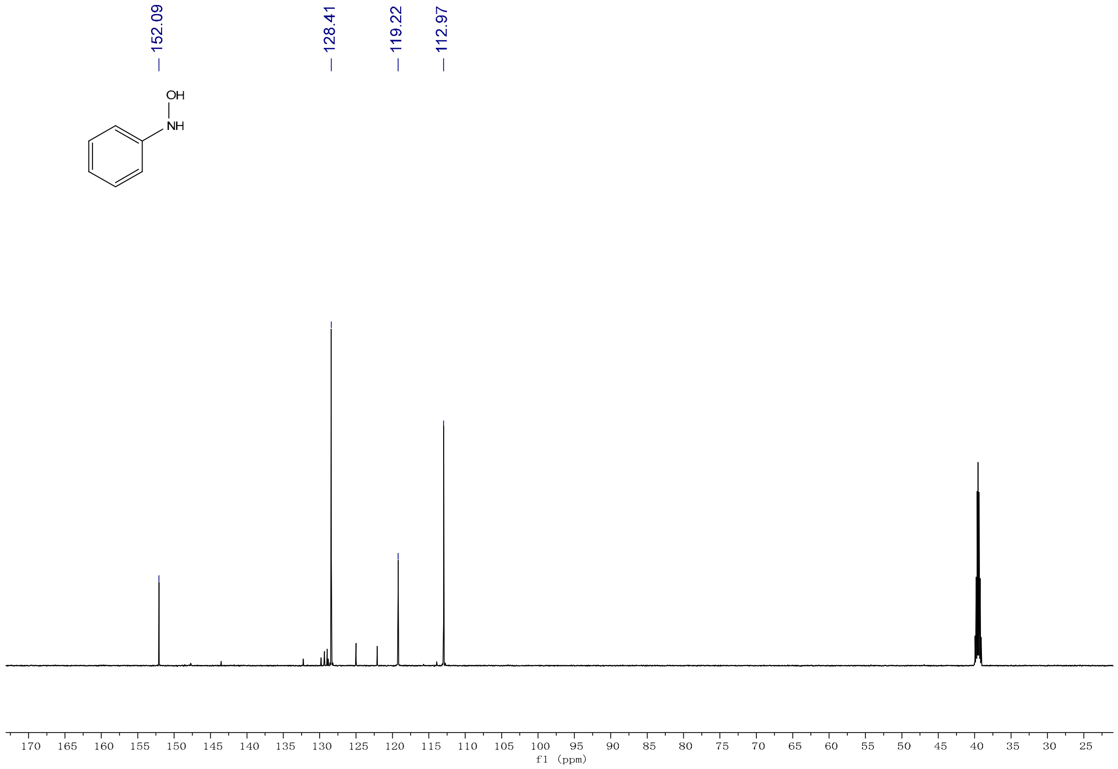

Figure S12. ^{1}H and ^{13}C NMR spectra of *N*-phenylhydroxylamine

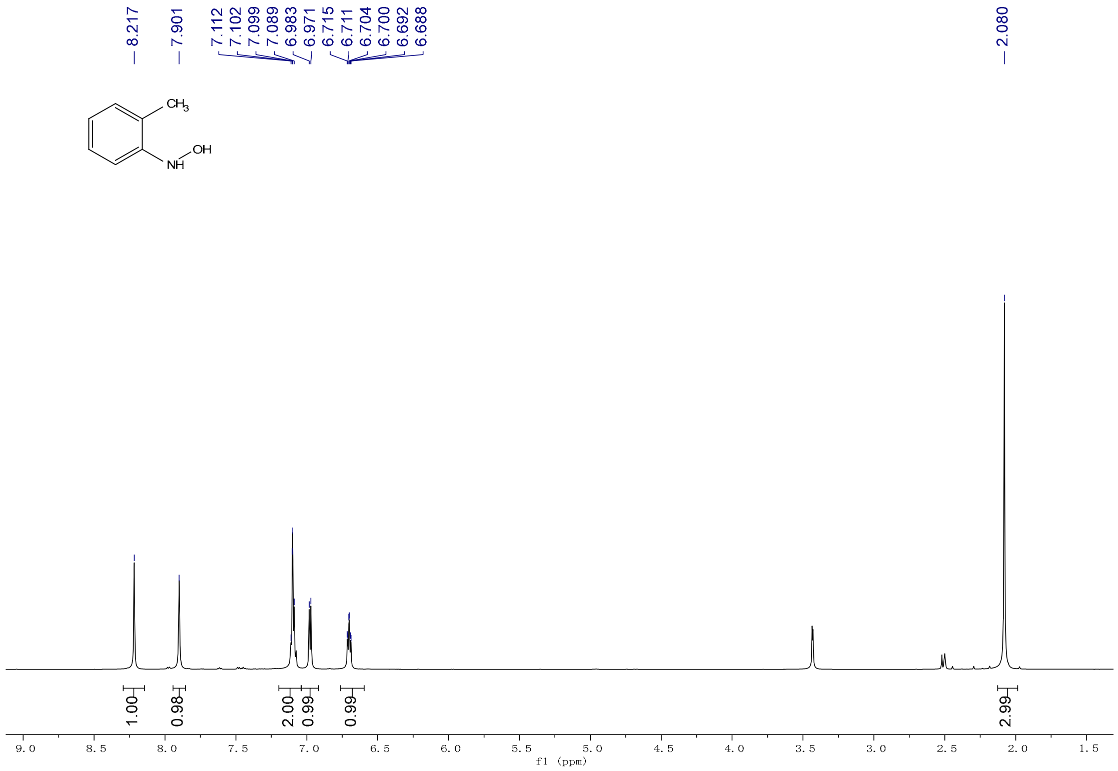

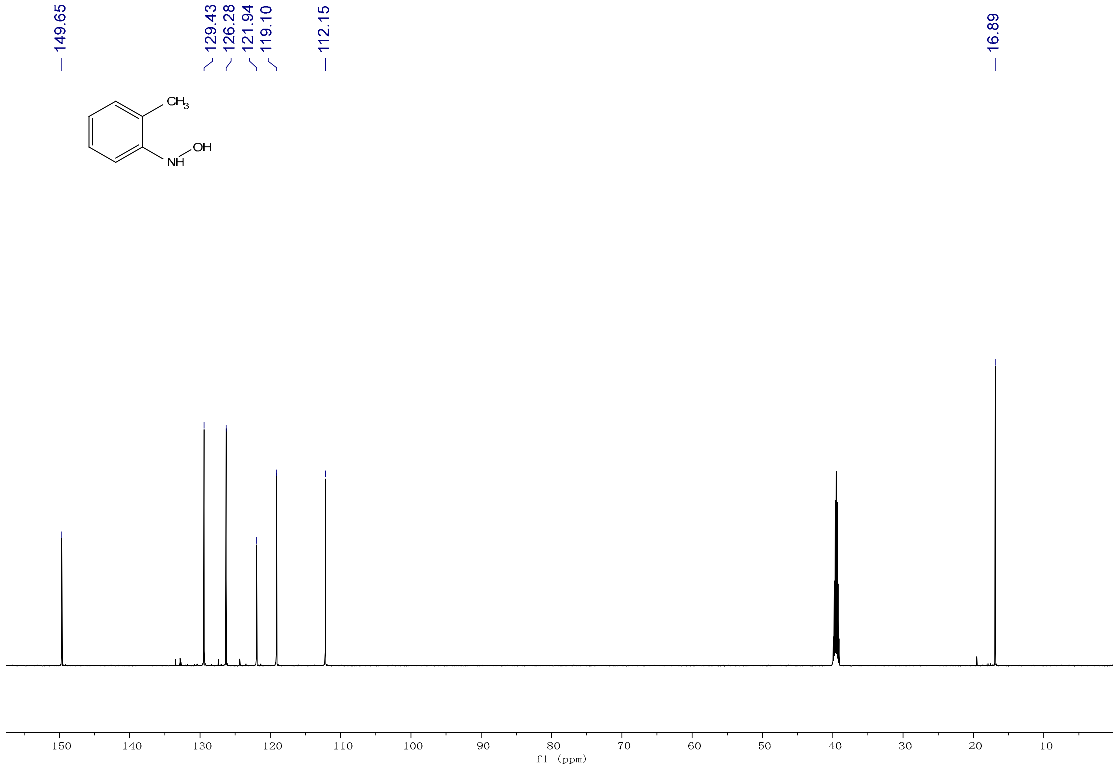

Figure S13. ^{1}H **and** ^{13}C **NMR spectra of *N*-(2-tolyl)hydroxylamine**

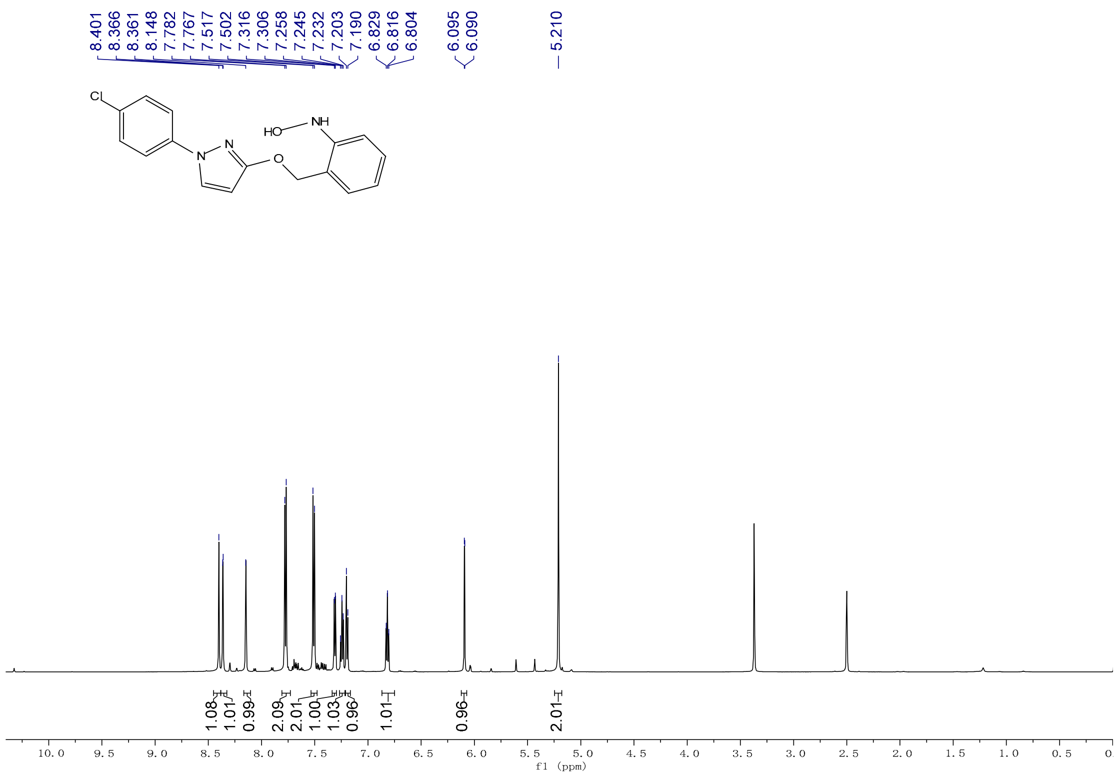

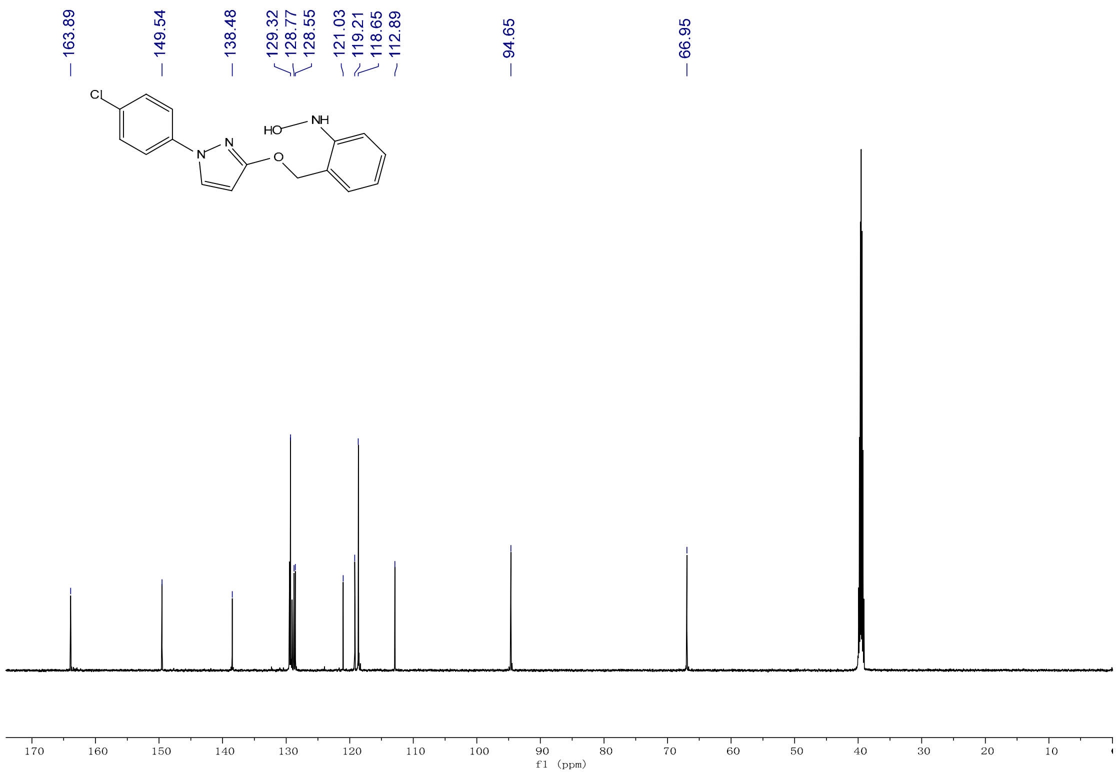

Figure S14. ^{1}H and ^{13}C NMR spectra of ***N*-(2-(((1-(4-chlorophenyl)-1H-pyrazol-3-yl)oxy)methyl)phenyl)hydroxylamine**

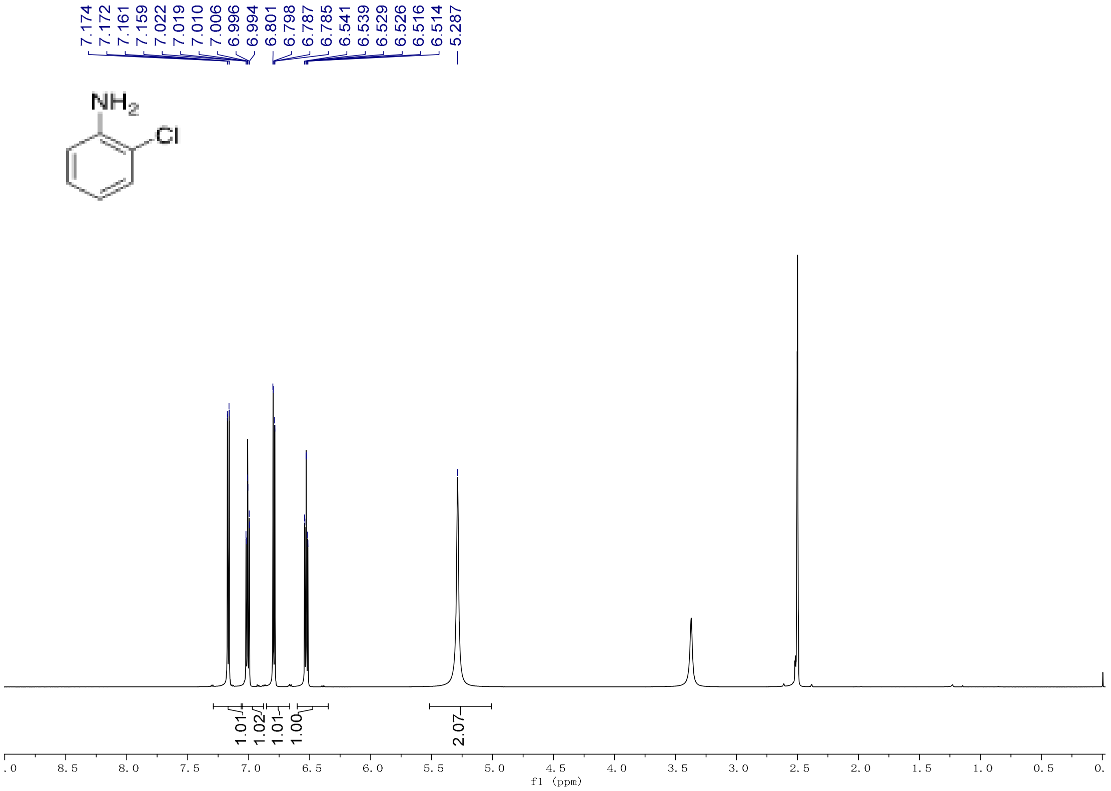

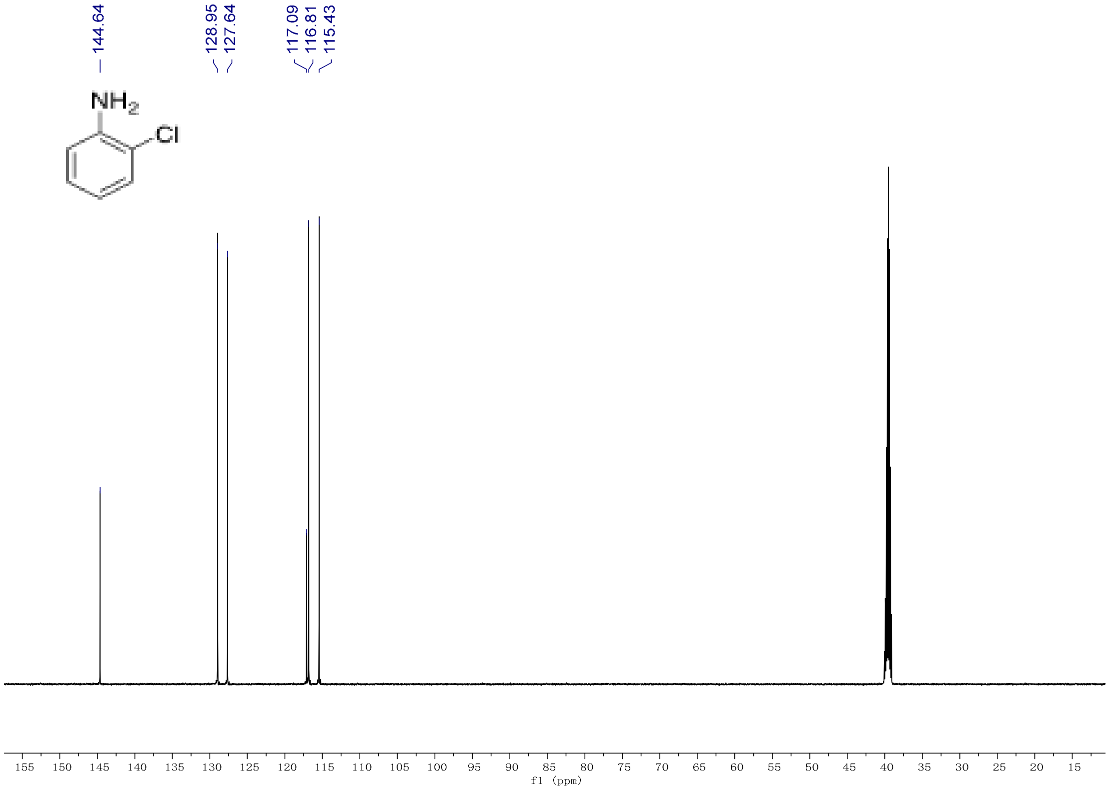

Figure S15. ^{1}H and ^{13}C NMR spectra of ***o*-Chloroaniline**

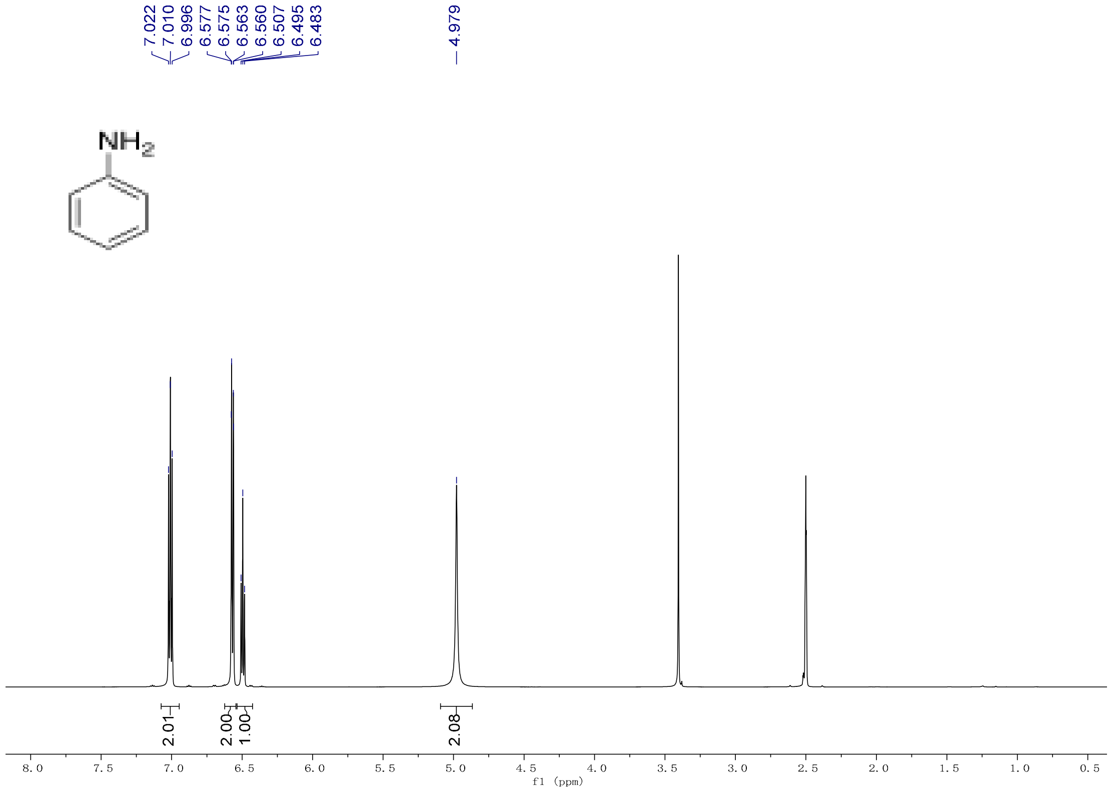

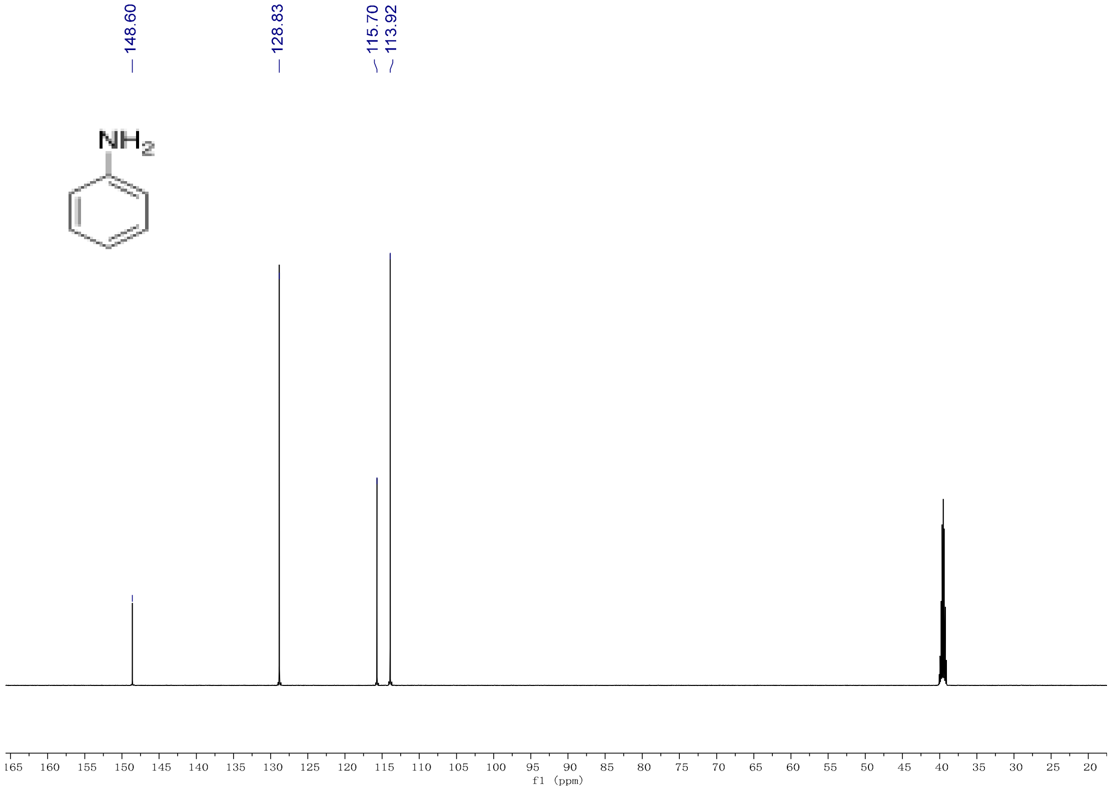

Figure S16. ^{1}H and ^{13}C NMR spectra of aniline

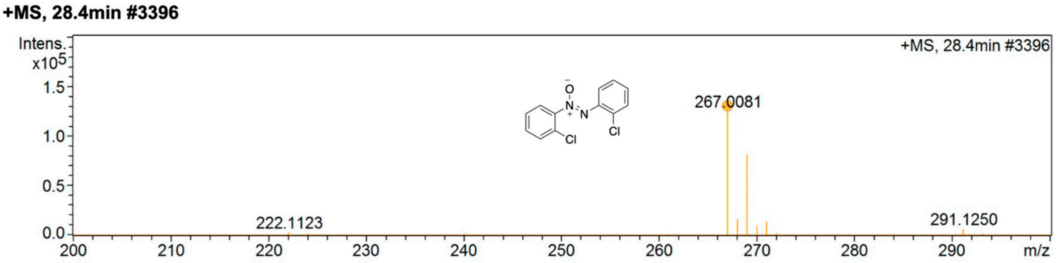

Meas. m/z	Ion Formula	m/z	err [ppm]	mSigma
267.0081	C12H9Cl2N2O	267.0086	1.9	3.6

Figure S17. LC-MS spectra of AZO

References

1 Doherty, S.; Knight, J.G.; Backhouse, T.; Summers, R.J.; Abood, E.; Simpson, W.; Paget, W.; Bourne, R.A.; Chamberlain, T.W.; Stones, R.; et al. Highly selective and solvent-dependent reduction of nitrobenzene to N-phenylhydroxylamine, azoxyben-zene, and aniline catalyzed by phosphino-modified polymer immobilized ionic liquid-stabilized AuNPs. *ACS Catal.* **2019**, *9*, 4777–4791. https://doi.org/10.1021/acscatal.9b00347.

2 Sharma, S.D.; Gogoi, P.; Konwar, S. A highly efficient and green method for the synthesis of 3,4-dihydropyrimidin-2-ones and 1,5-benzodiazepines catalyzed by dodecyl sulfonic acid in water. *Indian J. Chem. Sect. B Org. Chem. Incl. Med. Chem.* **2007**, *46B*, 1672–1678.

3 Chuang, H.Y.; Schupp, M.; Meyrelles, R.; Maryasin, B.; Maulide, N. Redox-Neutral Selenium-Catalysed Isomerisation of para-Hydroxamic Acids into para-Aminophenols. *Angew. Chemie-Int. Ed.* **2021**, *60*, 13778–13782.

4 Kallitsakis, M.G.; Ioannou, D.I.; Terzidis, M.A.; Kostakis, G.E.; Lykakis, I.N. Selective Photoinduced Reduction of Nitroarenes to N-Arylhydroxylamines. *Org. Lett.* **2020**, *22*, 4339–4343.

5 Xu, F.; Chen, J.L.; Jiang, Z.J.; Cheng, P.F.; Yu, Z.Q.; Su, W.K. Selective hydrogenation of nitroaromatics to N-arylhydroxylamines in a micropacked bed reactor with passivated catalyst. *RSC Adv.* **2020**, *10*, 28585–28594.
